# Saccadic scanpath length: an index for human threat conditioning

**DOI:** 10.3758/s13428-020-01490-5

**Published:** 2020-11-09

**Authors:** Yanfang Xia, Filip Melinscak, Dominik R. Bach

**Affiliations:** 1 University of Zurich, Lenggstrasse 31, CH-8032 Zurich, Switzerland; 2 University College London, London, UK

**Keywords:** Saccadic eye movement, Fear conditioning, Pavlovian conditioning, Attentional bias, Threat memory

## Abstract

Threat-conditioned cues are thought to capture overt attention in a bottom-up process. Quantification of this phenomenon typically relies on cue competition paradigms. Here, we sought to exploit gaze patterns during exclusive presentation of a visual conditioned stimulus, in order to quantify human threat conditioning. To this end, we capitalized on a summary statistic of visual search during CS presentation, scanpath length. During a simple delayed threat conditioning paradigm with full-screen monochrome conditioned stimuli (CS), we observed shorter scanpath length during CS+ compared to CS- presentation. Retrodictive validity, i.e., effect size to distinguish CS+ and CS-, was maximized by considering a 2-s time window before US onset. Taking into account the shape of the scan speed response resulted in similar retrodictive validity. The mechanism underlying shorter scanpath length appeared to be longer fixation duration and more fixation on the screen center during CS+ relative to CS- presentation. These findings were replicated in a second experiment with similar setup, and further confirmed in a third experiment using full-screen patterns as CS. This experiment included an extinction session during which scanpath differences appeared to extinguish. In a fourth experiment with auditory CS and instruction to fixate screen center, no scanpath length differences were observed. In conclusion, our study suggests scanpath length as a visual search summary statistic, which may be used as complementary measure to quantify threat conditioning with retrodictive validity similar to that of skin conductance responses.

## Introduction

Pavlovian threat conditioning (also termed fear conditioning) is widely used across species to investigate associative learning about aversive events. In this paradigm, initially neutral cues (conditioned stimuli, CS) are paired with aversive events, usually electrical shocks or white noise bursts (unconditioned stimulus, US). The creation of an implicit CS-US association is assessed by observation of the organism’s response during CS presentation. This includes overt behavior such as freezing (Bouton & Bolles, 1980; Roelofs, Hagenaars, & Stins, 2010), autonomic nervous system responses, measured as changes in skin conductance (Bach, Daunizeau, Friston, & Dolan, 2010; Staib, Castegnetti, & Bach, 2015), pupil size (Korn, Staib, Tzovara, Castegnetti, & Bach, 2017), heart period (Castegnetti et al., 2016) or respiration amplitude (Castegnetti, Tzovara, Staib, Gerster, & Bach, 2017), and the modulation of externally elicited behaviors, such as fear-potentiated startle (Blumenthal, 1988; Blumenthal et al., 2005; Khemka, Tzovara, Gerster, Quednow, & Bach, 2017) or Pavlovian-to-instrumental transfer (Xia, Gurkina, & Bach, 2019a). While all of these measures have specific advantages and disadvantages (see Ojala & Bach, 2020 for a review), a common limitation is their modest retrodictive validity. Retrodictive validity is the effect size to distinguish CS+/CS- trials, and is monotonically related to the accuracy (combined trueness and precision) of measuring the US expectation (Bach & Melinscak, 2020; Bach, Melinscak, Fleming, & Voelkle, 2020). This motivates the development of complementary measures, which in combination might serve to increase retrodictive validity, preferably without increasing measurement complexity. Here, we focused on gaze patterns which are recorded with the same eyetracking equipment used to record pupil dilation, an increasingly important threat conditioning measure (Korn et al., 2017; Tzovara, Korn, & Bach, 2018; Bach, Näf, Deutschmann, Tyagarajan, & Quednow, 2019).

Gaze patterns are influenced by top-down and bottom-up attention (Schutz, Braun, & Gegenfurtner, 2011; Theeuwes, 2010). The motivation for investigating gaze patterns in threat conditioning mainly derives from previous work investigating bottom-up processes. Various salient stimuli can capture covert (e.g., Mathews & MacLeod, 1985) and overt attention (see for review Schutz et al., 2011). Increased overt attention towards threat-conditioned cues has been demonstrated in various stimulus competition paradigms. CS+ are more often attended than CS- when presented simultaneously with an irrelevant neutral cue during conditioning (Austin & Duka, 2010; Koenig, Uengoer, & Lachnit, 2017). In a visual search task after conditioning with CS as distractor(s), CS+ attracted more (erroneous) saccades (Mulckhuyse, Crombez, & Van der Stigchel, 2013; Mulckhuyse & Dalmaijer, 2016; Koenig et al., 2017), dwell time on CS+ after such saccades was longer (Koenig et al., 2017), and time to reach (correct) target was longer during CS+ (Mulckhuyse & Dalmaijer, 2016; Nissens, Failing, & Theeuwes, 2017). Saccade trajectory deviation towards CS+ depended on saccade initiation latency (Mulckhuyse et al., 2013; Nissens et al., 2017) while saccade latency towards CS+ and CS- showed no difference in one study (Mulckhuyse & Dalmaijer, 2016). In an instructed saccade task after conditioning, saccades were faster towards CS+ (Schmidt, Belopolsky, & Theeuwes, 2015), and CS+ attracted more (erroneous) saccades (Schmidt et al., 2015; Hopkins, Helmstetter, & Hannula, 2016). It is currently unclear whether overt attention depends on uncertainty of the upcoming US (Hogarth, Dickinson, Austin, Brown, & Duka, 2008; Koenig et al., 2017) or on its aversive value (Wise, Michely, Dayan, & Dolan, 2019).

Despite overall good evidence in favor of a CS+ induced attention bias, we note that studies are somewhat heterogeneous in their dependent measures and results. Furthermore, in typical threat conditioning experiments, visual CS are presented on their own at a central location of the screen. In such a task, stimulus competition measures may not be ideally suited to capture overt attention.

**Fig. 1:**
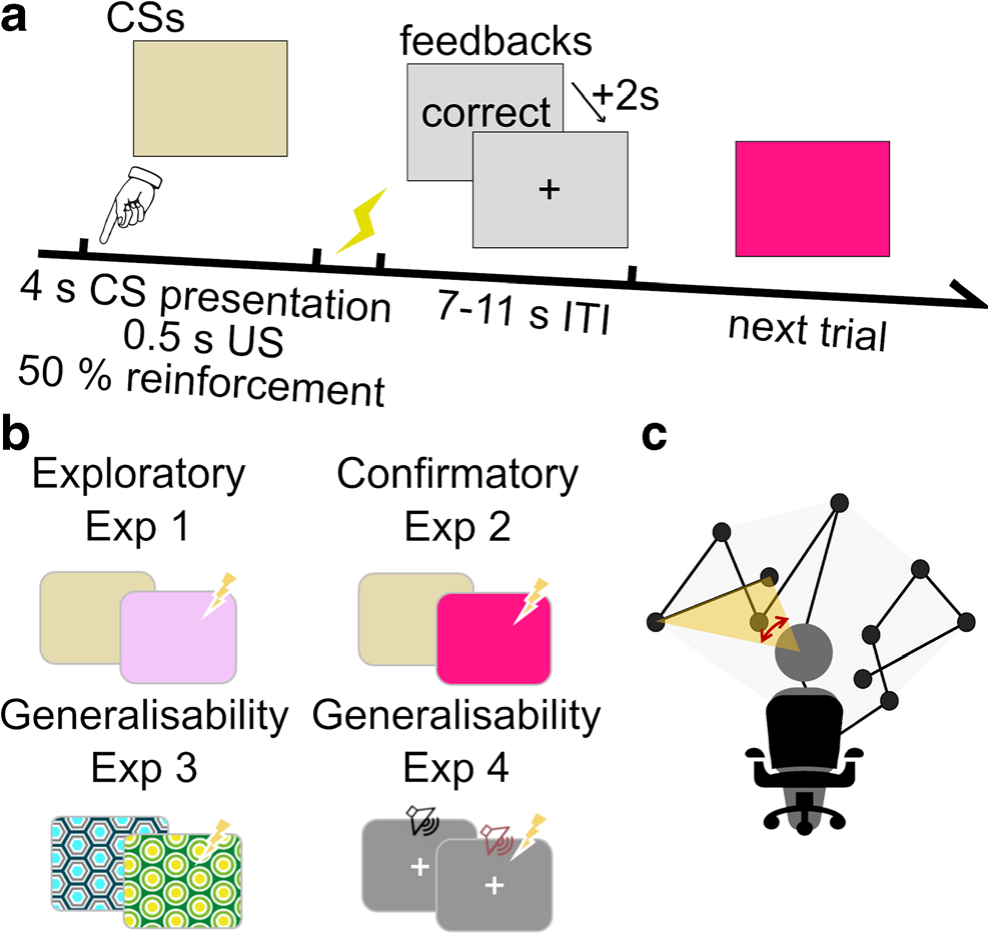
Experimental procedure and scanpath measure. **a** Pavlovian threat conditioning paradigm (details correspond to Exp 2). In each trial, a CS is presented for 3.5 (Exp 1) or 4 s (Exp. 2–4). Participants are asked to indicate CS by key press. In 50% of CS+ trials, CS presentation co-terminated with a 0.5-s electric shock. Response feedback (“correct”, “incorrect”, “only press RIGHT or LEFT”) is shown for 2 s at the beginning of ITI. A fixation cross is shown during the remaining ITI. **b** CS in the 4 experiments. **c** After linear interpolation of missing data points, scan speed was calculated as the central angle between two adjacent gaze data points, divided by their difference in time (*yellow angle as measured by the red bi-directed arrow*). Integrating this over time yielded scanpath length. *CS* conditioned stimulus, *US* unconditioned stimulus, *ITI* inter-trial interval

Hence, the primary focus of the current study was to develop a complementary index of overt attention to distinguish gaze patterns during CS. We based this on a metric of visual search, namely scanpath length. We used a previously published experiment as discovery data set (Table 1 and Fig. 1, Experiment 1), where we investigated whether and how scanpath length was affected by presentation of full-screen pure colors as CS (without fixation cross) in a delayed discriminant Pavlovian threat conditioning paradigm. We replicated these results in an independent previously published data set, as well as in a third data set specifically recorded for this purpose. Finally, we investigated whether scanpath length also differentiates auditory CS under instructed fixation.

**Table 1:** Experimental configuration and recordings

Experiment (Data set code)	CS	SOA	ITI	Experimental settings	Fixation cross	Measures analyzed	Reference and data availability
Exploratory Exp 1 (PIT1)	visual	3.0	2.5 s	threat memory acquisition with 16 CS+US+, 16 CS+US- and 32 CS-US-	only in ITI	SCR, Pupil size, Gaze	Xia et al., 2019a, b, c Data set DOI: 10.5281/zenodo.2641734
Confirmatory Exp 2 (PIT2)	visual	3.5	7-11 s	threat memory acquisition with 16 CS+US+, 16 CS+US- and 32 CS-US-	only in ITI	SCR, ECG, Pupil size, Gaze	Xia et al., 2019a, b, c Data set DOI: 10.5281/zenodo.2641738
Generalizability Exp 3 (ViS)	visual	3.5	7-11 s	threat memory acquisition with 30 CS+US+, 30 CS+US- and 60 CS-US- in 2 blocks threat memory extinction with 20 CS+US- and 20 CS-US-	only in ITI	SCR, ECG, Gaze	Data set DOI: 10.5281/zenodo.3667715
Generalizability Exp 4 (PubFe)	auditory	3.5	7, 9, 11 s	threat memory acquisition with 40 CS+US+, 40 CS+US- and 80 CS-US-	always	SCR, ECG, Pupil size, Gaze	Korn et al., 2017 Data set DOI: 10.5281/zenodo.1168494

In Exp 1, the visual CS were presented for 3.5 s, whereas in Exp 2 and 3, CS were presented for 4.0 s. Half of CS+ trials were reinforced by US delivery during 0.5 s before CS offset which co-terminated with CS (CS+US+), and the other half were not (CS+US-). In Exp 4, grey background with a fixation cross in the center was always presented on the screen. CS were two 50-ms sine tones and the 0.5-s US was delivered 3.5 s after CS delivery in half of CS+ trials. Trial order in all experiments was randomly balanced over the entire experiment, or per block in Exp 3. Inter-trial interval was 2.5 s in Experiment 1 and was randomly determined to be an integer between 7 and 11 s in Experiment 2 and 3; in Exp 4, ITI was randomly drawn from {7 s, 9 s, or 11 s}. CS, conditioned stimulus; US, unconditioned stimulus; SOA, stimuli onset asynchrony; ITI, intertrial interval; SCR, skin conductance responses; ECG, electrocardiography. Only CS- and CS+US- trials were analyzed.

In doing so, our primary criterion was retrodictive validity, which jointly assesses the trueness of the measurement (i.e., how close the average over many measurements is to the true score) and its precision (i.e., how much does the measurement vary when true score is constant) (Bach et al., 2020). We also report an approximated split-half reliability. Because reliability is only meaningful if the measure is valid in the first place (Cronbach & Meehl, 1955), and strongly depends on the presence of interindividual variability in learning the CS-US association (Brandmaier et al., 2018), we do not use it as a development criterion.

## Method

### Participants

We recruited four independent groups of healthy participants with normal or corrected-to-normal vision from general population. The study, including the form of taking written informed consent, was conducted in accordance with the Declaration of Helsinki and approved by the governmental research ethics committee (Kantonale Ethikkommission Zürich: KEK-ZH-2013-0118).

Participants without unconditioned (skin conductance) response (UR) to US (electric shock), and those who did not follow task instructions were excluded. This excluded one participant (no UR) in Experiment 1, three (two no UR, one did not follow instructions) in Experiment 2, and three (two no UR and one did not follow instructions to sit still) in Experiment 3. We thus report data from 21 participants in Experiment 1 (12 females, mean age ± standard deviation 26.4 ± 3.6 years), 35 participants in Experiment 2 (21 females, 24.7 ± 3.8 years), 26 participants in Experiment 3 (15 females, 24.6 ± 3.0 years), and 22 participants in Experiment 4 (15 females, 26.4 ± 5.2 years). Experiments 1–2 were primarily conducted to assess Pavlovian-to-Instrumental transfer after threat conditioning; behavioral and psychophysiological data from these experiments are published in a previous report (Xia et al., 2019a; see for data sets: Xia, Gurkina, & Bach, 2019b, 2019c). Experiment 4 had been conducted during development of a model for threat-conditioned pupil size responses (Korn et al., 2017); psychophysiological results from this experiment are also published (see for data set: Korn, Staib, Tzovara, Castegnetti, & Bach, 2018).

### Stimuli and apparatus

#### Psychophysiological recording

All experiments were conducted in a dark and soundproof chamber. Participants’ heads were positioned on a chin rest in front of the monitor (Dell P2014h, 20” set to an aspect ratio of 4:3 at 60 Hz, and a resolution of 1152 x 864 in Experiments 1, 2, and 3; Dell P2012h with same settings in Experiment 4) with a distance of 700 mm from head to monitor. Gaze direction coordinates in pixels were collected for both eyes with an EyeLink 1000 System (SR Research, Ottawa, ON, Canada) at a sampling rate of 500 Hz, with a horizontal distance of 470 mm between eyes and eye-tracker. Skin conductance responses (SCR) were recorded from the thenar/hypothenar of non-dominant hand by two 8-mm disk Ag/AgCl cup electrodes (EL258, Biopac Systems Inc., Goleta, CA) and 0.5% NaCl gel (GEL 101, Biopac Systems Inc., Hygge & Hugdahl, 1985), with an SCR coupler/amplifier (V71-23, Coulbourn Instruments). Electrocardiogram (ECG) data was collected with four 45-mm, pregelled Ag/AgCl adhesive electrodes attaching to the outsides of wrists and ankles, respectively. The ECG configuration yielding the clearest R spikes was identified visually before every experiment, and recorded. Data time series of SCR and ECG were digitized at 1000 Hz (DI-149, Datag Inc., Akron, OH) and collected with Windaq software (Dataq Inc.).

#### Unconditioned stimulus

US was a 500-ms train of 250 square electric pulses with a duty cycle of 10%, and was delivered through a pin-cathode/ring-anode configuration with a constant current stimulator (Digitimer DS7A, Digitimer, Welwyn Garden City, UK) on participants’ dominant forearm. The intensity of US was estimated in a two-phase procedure: 1) staircase testing phase to determine the pain threshold by delivering a series of gradually strengthened shocks from unperceivable to clearly painful level; 2) random test of 14 stimuli below this value which were rated on a scale from 0% (no sensation) to 100% (clearly painful). The final intensity used during experiments corresponded to 85% of the initial pain threshold and was derived from a linear interpolation of the ratings.

### Experimental setup

#### Common settings

All four experiments were presented with Cogent 2000 Toolbox (v1.32, www.vislab.ucl.ac.uk) in MATLAB (2012b, The MathWorks, Natick, MA, USA). All experiments used a delay threat conditioning procedure with two CS, one of which co-terminated with US in a 50% reinforcement schedule (CS+) and the other never co-terminated with US. Table 1 summarizes experimental configurations. We asked participants to press one of two designated keys for each of CS. Participants were explicitly instructed that their key press response did not influence US delivery and US delivery was only related to preceding CS, but they were not informed about the CS-US contingency or about the number of CS+/-. Assignment of CS+/- to physical CS properties was counterbalanced across participants. Response key/CS association was counterbalanced in Exp 1-2 and randomly determined in Exp 3–4. Figure 1 shows an example of experimental procedure together with CS used in each experiment.

#### Exploratory Experiment 1 – Simple visual CS (Data set code: PIT1)

CS were two monochrome colors (Fig. 1b) presented full-screen (light purple with RGB values 0.9510, 0.7741, 0.9759, and light yellow, RGB 0.8970, 0.8576, 0.6874). The experiment consisted of 64 trials in random order: 16 CS+ followed by US (CS+US+), 16 CS+ not followed by US- (CS+US-), and 32 CS-. No fixation cross was presented during CS. During ITI, a black fixation cross was presented in the center of a grey background (RGB 0.85, 0.85, 0.85). Participants were instructed to keep looking at the screen throughout.

#### Confirmatory Experiment 2 – Simple visual CS (Data set code: PIT2)

Procedure was the same as in Exp 1 but with different CS colors (light yellow with RGB 0.8970, 0.8576, 0.6874, and rose pink with RBG 1, 0.0745, 0.5216) to enhance discriminability, and different CS and ITI timing as detailed in Table 1.

#### Generalizability Experiment 3 – Simple visual CS (Data set code: ViS)

Experiments 1–2 assessed spontaneous eye movements in the absence of any visual information on the monochrome screen, and without any instruction to fixate or visual cues aiding fixation. Experiments 3 was designed to investigate the generalizability of these findings to a situation where a regular screen pattern provides information but also aids fixation. Furthermore, we included an extinction phase. CS were two full-screen patterns, with approximately equal brightness, contrast, and spatial frequencies. There were two blocks of the acquisition phase with 15 CS+US+, 15 CS+US-, and 30 CS- in each block, and an extinction phase with 20 CS- and 20 CS+ trials without US delivery, presented in one single block. The order of trials in each block was randomized. The three blocks were separated by brief self-paced breaks. No fixation cross was presented during CS. During ITI, a black fixation cross was presented in the center of a grey background (RGB 0.7, 0.7, 0.7). Participants were instructed to keep looking at the screen throughout.

#### Generalizability Experiment 4 – Simple auditory CS (Data set code: PubFe)

Experiment 4 was selected to investigate the generalizability of our findings to a situation with clear instruction to fixate, and non-visual CS. CS were two sine tones with constant frequency (220 Hz or 440 Hz, 50-ms onset and offset ramp). Sound stimuli were delivered by headphones at approximately 60 dB (HD 518, Sennheiser, Wendemark-Wennebostel, Germany). During the entire task, a white fixation cross was presented on a grey background. There were 40 CS+US+, 40 CS+US-, and 80 CS- trials in two sessions with a brief self-paced break in-between.

### Data analysis

#### Eye-tracker data pre-processing

All data were analyzed in MATLAB (2015b, The MathWorks, Natick, MA, USA) with standard routines in PsPM (4.0, bachlab.github.io/PsPM/) and custom-written code. Eye-tracker data were imported into PsPM after excluding blanks (i.e., saccades and blinks). Gaze coordinates in pixels, of the eye with fewer missing values, were extracted, linearly interpolated, and converted into Cartesian coordinates in millimeters, with the screen center as origin, and right and upward as positive. Assuming that participants' nasion was approximately located on a straight line through the screen center and perpendicular to the screen, we then projected (x, y)-coordinates into a three-dimensional Cartesian coordinate system with the participants nasion as origin such that every gaze point on the screen had a coordinate of (x, y, z) with z = 700. We then converted these coordinates into a spherical coordinate system. The azimuthal angle *θ*, a counterclockwise angle in the horizontal x-z plane with *θ = 0* in the positive x-axis and *θ* = π/2 for straightforward gaze, was then calculated with the following identity:

$$ \theta =\left\{\begin{array}{c}\arctan \left(\frac{z}{x}\right),x\ne 0;\theta \in \left[0,\frac{\pi }{2}\right[\cup \left]\frac{\pi }{2},\pi \right]\ \\ {}\frac{\pi }{2},x=0\end{array}\right.; $$

and the elevation angle *φ* from x-z plane, was calculated as

$$ \varphi =\arctan \left(\frac{y}{\sqrt{x^2+{z}^2}}\right);\varphi \in \left]-\frac{\pi }{2},\frac{\pi }{2}\right[. $$

As the radial distance contains no further information, it was discarded. These two angles were calculated with the MATLAB function *cart2sph,* which uses the identities above. The unit of angles was then converted from radians into angular degrees. The central angle ∆*λ* (identical to the length of the great circle arc between the two points on a unit sphere) between each of two adjacent gaze data points was subsequently computed with the MATLAB (Mapping toolbox) function *distance* using Haversine formula (note that at this stage, missing values due to saccades are already interpolated):

$$ a={\mathit{\sin}}^2\left(\frac{\varphi_2-{\varphi}_1}{2}\right)+\cos \left({\varphi}_1\right)\cdotp \cos \left({\varphi}_2\right)\cdotp {\mathit{\sin}}^2\left(\frac{\theta_2-{\theta}_1}{2}\right); $$

$$ \Delta  \lambda =2\arctan \left(\frac{\sqrt{a}}{\sqrt{1-a}}\right),a\in \left[0,1\right[; $$

**Fig. 2:**
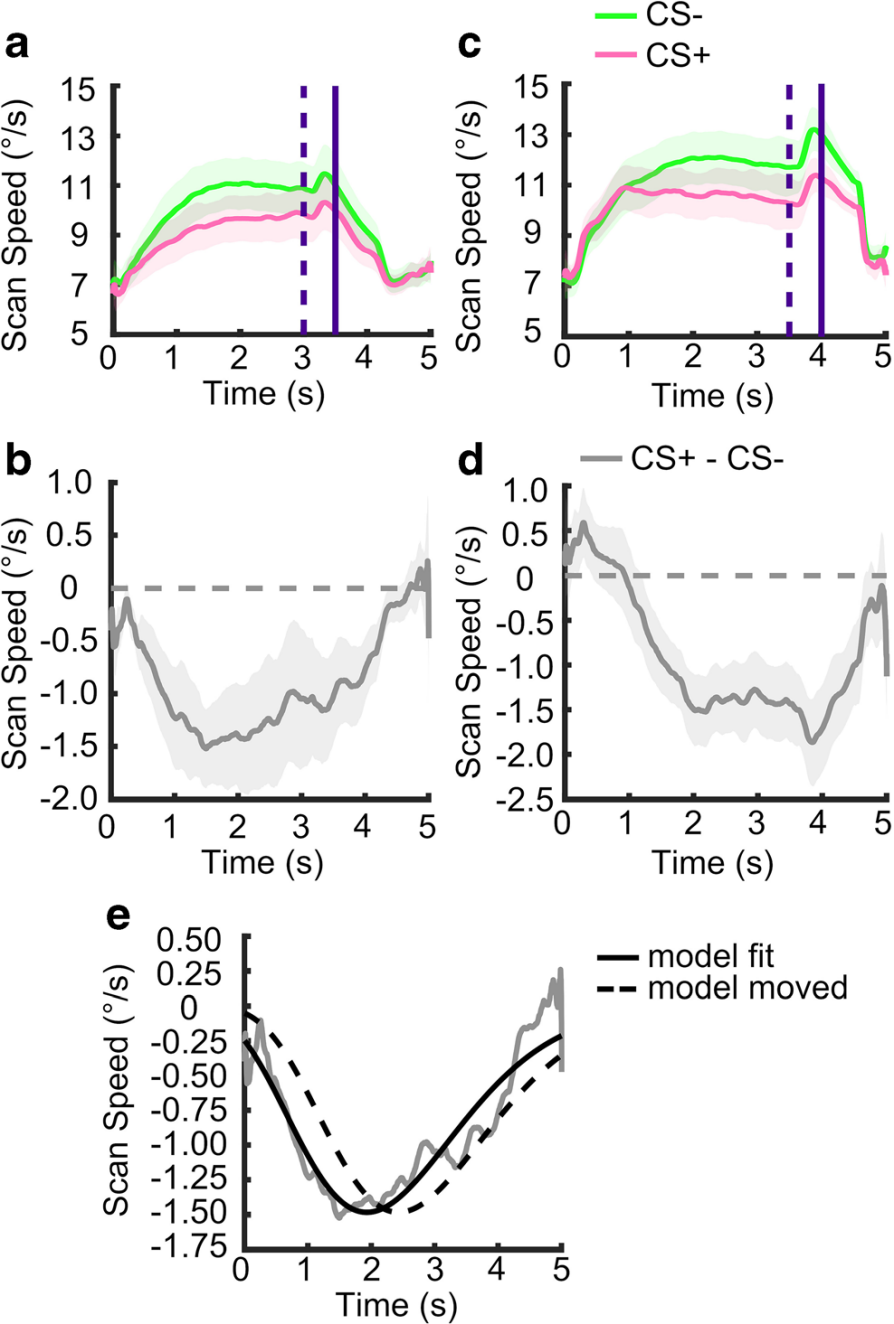
Time series of scan speed. Only non-reinforced (US-) trials were analyzed. **a** Time series of scan speed in Exp 1. *Dashed* and *solid vertical lines* represent the onset of US and the end of a trial, respectively. **b** Difference of scan speed time series between CS+ and CS- in Exp 1. **c–d** Time series of scan speed and difference time series in Exp 2. **e** Empirical response function. A gamma probability density function (*solid black curve*) was fitted to scan speed time series from Exp 1 (*solid grey curve*). This model was then moved 0.5 s to the right to account for the later US in subsequent experiments (*dashed black curve*). Data shown in panels **a–d** are grand means, i.e., responses averaged across first over trials and then over participants ± SEM (computed over participants after trial averaging, *shaded regions*)

This central angle was regarded as scanpath length between two adjacent gaze points and then converted to scan speed (degree/sec). No filtering was applied for statistical analysis. For visualization and development of the response model, the time series of scan speed data (Fig. 2) was averaged across trials in each condition for each participant, then smoothed with MATLAB function *smooth* with a span of 501, corresponding to 1002 ms. This smoothing window provided the clearest visualization of time series. Using smaller smoothing windows (50 ms, 102 ms) and lowpass filters (5, 10, 20, and 50 Hz) resulted in very similar response function, which in turn led to almost identical parameter estimates in general linear convolution model (GLM) inversion (see below). The smoothed data were then averaged across participants and plotted. SEMs of the plots were calculated across participants using the smoothed data. Notably, due to our method of interpolating missing values, responses to the US can affect scanpath speed even before the US occurs. This is why we excluded data from US+ trials for all statistical analyses.

#### Total scanpath length in different time windows

To compute total scanpath length we integrated scan speed over different time windows before anticipated US onset. For Exp. 1–2, we used time windows in 0.5-s steps. For Exp 3–4, we only extracted scanpath length for the optimal time window. This measure was then averaged across all CS-, or CS+US- trials within participants.

#### Empirical response function and model inversion

A linear time-invariant system was used to model the difference of anticipatory scan speed elicited by CS+US- versus CS- as in previous work (Castegnetti et al., 2016, 2017; Korn et al., 2017). The difference of grand means of scan speed time series between CS- and CS+US- from Exp 1 was used to fit an empirical response function. In a heuristic function search, a gamma probability density function seemed to provide a good approximation:

$$ y=\frac{A}{\upmu^{\mathrm{k}}\Gamma (k)}{\left(t-{t}_0\right)}^{k-1}{e}^{-\frac{t-{t}_0}{\mu }}; $$

where y is the input scan speed, *t* is time, Γ is the gamma function, and *t*_*0*_ (onset latency), *k*, *μ*, and A are free parameters. These parameters were estimated with the MATLAB function *fminsearch* using the Nelder–Mead algorithm and ordinary least squares minimization (Lagarias, Reeds, Wright, & Wright, 1998).

This function was then used as a response function in a general linear convolution model (GLM) to estimate the amplitudes of observed scan speed responses. The GLM was written, for each participant as

$$ Y= X\beta +\epsilon; $$

where *Y* is observed data time series, *X* is a design matrix constructed by convolving a stick function for each CS onset with the response function, *β* represents the amplitude of the scan speed responses, and *ϵ* is independent and identically distributed noise. GLM implementation was the same as for our previous psychophysiological models (Bach, Castegnetti, Korn, Gerster, Melinscak, and Moser, 2018a).

#### Fixation distribution

For each participant, we extracted gaze coordinates and fixation duration from CS onset to US onset in all CS- and CS+US- trials, which we plotted as heat maps (gaze coordinates) and histograms (fixation duration).

#### Psychophysiological data pre-processing and analysis (Experiment 3)

SCR data were visually inspected, and one participant excluded due to electrode detachment. SCR data were then filtered with a 1^st^-order bidirectional band-pass Butterworth filter (cut-off frequencies: 0.0159–5 Hz), and down-sampled to 10 Hz. Resulting time series were analyzed by non-linear inversion of a PsPM that describes the anticipatory and evoked SCR under a canonical response function (Bach, Flandin, Friston, & Dolan, 2009; Bach et al., 2010; Staib et al., 2015; Gerster, Namer, Elam, & Bach, 2018). Specifically, a fixed-latency response at CS onset and a fixed-latency response at (potential) US onset were estimated for each trial. The inversion algorithm was not informed about trial type or the presence of an US. We included only non-reinforced trials into statistical analysis to avoid any contamination by US responses. We note that in all four experiments, CS-US intervals (stimuli onset asynchrony, SOA) were relatively short (3–3.5 s). The inversion algorithm used for SCR analysis was developed and implemented specifically for such short (up to 4 s) CS-US intervals and accounts for overlapping in SCR (Staib et al., 2015). Next, we detected R-spikes in the ECG using a modified Pan–Tompkins algorithm implemented in PsPM (Castegnetti et al., 2016; Paulus, Castegnetti, & Bach, 2016). Inter-beat interval was mapped onto the time point of the following R spike, and values outside 600 ms and 1000 ms (corresponding to a heart rate outside 60–100 bpm) were excluded. Heart period was then linearly interpolated with 100-Hz sampling frequency and filtered with a 4th-order bidirectional band-pass Butterworth filter (cut-off frequencies: 0.015–0.5 Hz). To estimate the anticipatory heart period (bradycardia) response, we used a condition-wise general linear convolution model implemented in PsPM (Castegnetti et al., 2017).

### Statistical analysis

Statistical analysis was done in MATLAB (2015b and 2018b, The MathWorks, Natick, MA, USA) and R (v. 3.6.1, www.r-project.org). Due to our data pre-processing and response estimation procedures, the presence of a US can influence the estimate of CS-induced responses. For this reason, we discarded all CS+US+ trials as in previous work. To compare dependent measures between CS+US- and CS-, we used two-tailed paired *t* tests. Effect size was quantified as Hedge’s *g* using the following formula (Hedges & Olkin, 2014; Lakens, 2013):

$$ g=\mathrm{J}\left(n-1\right)\ast \frac{\overline{Xdiff}}{SDdiff}\ \mathrm{where}\ J(a)=\frac{\Gamma \left(\frac{a}{2}\right)}{\sqrt{\frac{a}{2}}\ \Gamma \left(\frac{a-1}{2}\right)}. $$

The corresponding confidence interval was computed using *bootci* in MATLAB (bias-corrected and accelerated bootstrapping). We also computed Akaike Information Criterion (AIC) as in previous work (Xia et al., 2019a). Log Bayes factor (LBF) was then calculated from AIC values by formula LBF = (AIC - AIC_ref_)/2. The number of free parameters in the calculation of AIC is the same for all methods), such that LBF are equal to those based on Bayesian Information Criterion. A two-tailed two-sample Kolmogorov–Smirnov test was used to compare distributions of fixation duration between CS- and CS+US- using *ks.test* in R.

### Reliability analysis

We also sought to address internal consistency of the scanpath metric and its correlation with other metrics of threat conditioning. This assesses whether an individual’s estimated place in the distribution of CS-US association strength is stable within the learning session. As a caveat, reliability is only meaningful if the measure is valid in the first place (Cronbach & Meehl, 1955). Furthermore, reliability depends not only on the precision of the measurement, but also stable variability in (true) learning between participants (Brandmaier et al., 2018). Typical experimental procedures are designed to reduce such variability (Hedge, Powell, & Sumner, 2018). In an ideal case of a threat conditioning experiment in which all participants learn CS-US association perfectly, but there is some (even small) measurement noise, any reliability metric will be zero, independent of the magnitude of measurement noise (Brandmaier et al., 2018).

In deriving a reliability metric, we had to account for the fact that learning is a dynamic process and change in the learning metric is expected from one trial, or learning session, to the next. Some consistency metrics (such as Cronbach’s alpha or intra-class correlation coefficients) would require single-trial measurements, which are not available for HPR; furthermore, some of these treat all data points as coming from the same population. To approximate internal consistency, we used a split-half procedure. We split the data, for each condition, into pairs of consecutive trials, under the assumption that true CS-US association within these pairs of trials was relatively similar. We randomly selected one of these trials into trial set 1, and the other into trial set 2, and computed a CS-US association strength metric for both sets of trials (by averaging single-trial estimates, and for HPR by computing a GLM with two regressors per condition). We then computed the correlation of the two partitions in terms of CS+ - CS-, for each measurement. We repeated this procedure 5000 times and computed the average of all correlations for each measurement. We note that this metric is constrained by the dynamic update of CS-US associations on every trial, and can reach high values only after learning asymptotes to stable values, which we did not expect to be the case in our experiment.

### Data and code availability

All data sets are available in anonymized form on www.zenodo.org (Table 1). Analysis code is available from OSF (10.17605/OSF.IO/U4GRC). Preprocessing methods are implemented in the most recent version of PsPM (https://github.com/bachlab/PsPM/releases).

## Results

We first confirmed that participants learned the CS-US contingencies during the acquisition phase in all four experiments. We note that due to our interpolation, filtering, and model inversion procedures, apparent CS responses in US+ trials can be influenced by unconditioned responses. This is why we did not analyze reinforced CS+US+ trials; the abbreviation CS+ henceforth refers to CS+US- trials. We contrasted CS+/CS- in several established threat conditioning measures. For Experiments 1–2 and 4, SCR, PSR, and (for Experiment 2) HPR were reported previously. These measures significantly differed between CS+/CS- in all experiments, with the exception of SCR in Experiment 4 (t(12) = 2.03, *p* = .065, g = 0.53), which was notably based on a small sample of 13 participants due to missing data. Similarly for Experiment 3, SCR and HPR differed between CS+/CS− (SCR mean ± SEM: 0.27 ± 0.04 μS vs. 0.23 ± 0.03 μS; t(24) = 3.28, *p* = .003; *g* = 0.64; and HPR 10.72 ± 3.87 ms vs. – 11.46 ± 3.63 ms; t(25) = 3.76; *p* < .001; *g* = 0.72).

### Experiment 1: Scan speed time series and trial summary statistics

Next, we analyzed the time series of scan speed during CS+ and CS- presentation in experiment 1. As visible in Fig. 2a, during both CS+ and CS-, scan speed was increased after CS onset, and decayed after US offset. Scan speed increased more during CS- than CS+ (Fig. 2a). This difference is depicted in Fig. 2b.

To derive a single summary statistic of scan speed over the entire trial, we first integrated from CS onset to US onset. This scanpath length measure was significantly different between CS+ and CS-(t(20) = 2.98; *p* = .007, paired *t* test; see LBF and effect size in Table 2).

**Table 2:** Comparison of different scan path length time windows, and GLM-derived response estimate for Exp 1 and 2

	Exploratory Exp 1							
		3.0 s	2.5 s	2.0 s	1.5 s	1.0 s	0.5 s	GLM
*df*		20	20	20	20	20	20	20
LBF		0.00	0.18	– 0.17	2.32	3.81	5.71	– 0.48
*|g|*		0.63	0.62	0.63	0.51	0.43	0.30	0.65
	Confirmatory Exp 2							
	3.5 s	3.0 s	2.5 s	2.0 s	1.5 s	1.0 s	0.5 s	GLM
*df*	34	34	34	34	34	34	34	34
LBF	0.00	– 1.53	– 4.85	– 6.69	– 4.86	– 0.91	1.42	– 5.27
*|g|*	0.47	0.52	0.62	0.68	0.62	0.50	0.41	0.63

Time windows are defined as period before US onset. CS-US intervals were 3.0 s and 3.5 s in Exp 1 and 2, respectively. Log Bayes factors (LBF, smaller is better) is calculated using the entire CS-US interval as reference model. Effect size is stated as Hedge’s *g*. GLM, general linear model

**Fig. 3:**
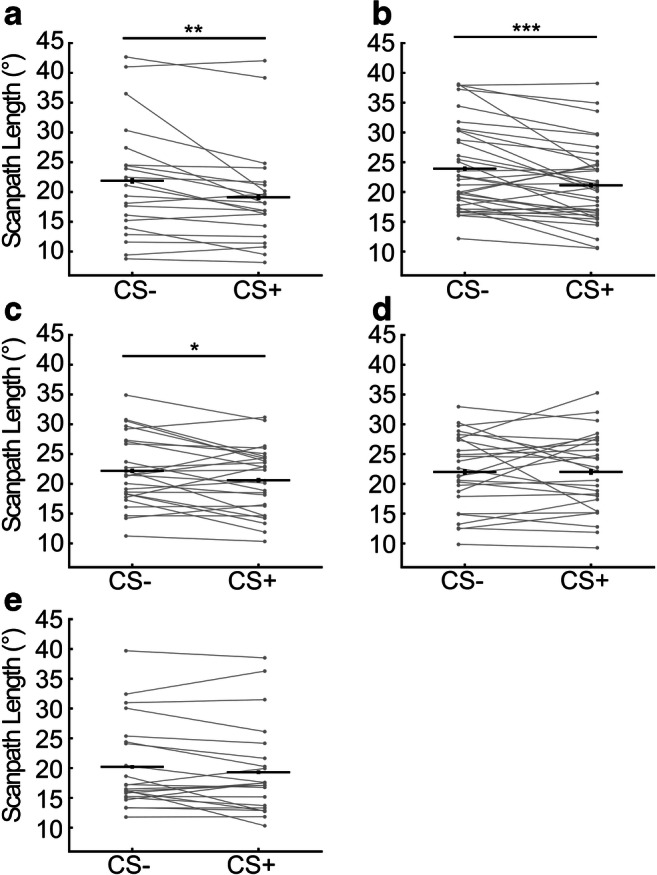
Total scanpath length in a 2-s period before US onset in **a** Exp 1, **b** Exp 2, **c** Exp 3 acquisition phase, **d** Exp 3 extinction phase, and **e** Exp 4. Only non-reinforced (US-) trials were analyzed. Inference statistics for Experiment 1 are presented for illustration only as the time window was optimized on data from this experiment. *Grey lines*: individual participant data. *Horizontal black lines*: mean. *Vertical black lines*: standard error of the CS+/CS- difference (Cousineau, 2005). * *p* < .05, ** *p* < .01, *** *p* < .001

Since scan speed appeared to best differentiate between CS- and CS+ after around 1–2 s into CS presentation until US onset, we sought to optimize this summary statistic. To avoid circular inference, we do not interpret inferential statistics for this optimization. Instead, we sought to confirm generalizability of the results in independent Experiment 2. We computed scanpath length over different time windows during CS presentation. Table 2 shows effect sizes. Among the time windows considered, the optimal time window to distinguish CS valences was a 2-s time period before US onset, where effect size to distinguish CS+/CS- was slightly, but according to model evidence, not decisively higher than for scanpath length computed over the entire CS-US interval (Fig. 3a, Table 2).

We sought to further optimize this measure by taking into account the shape of the scan speed time series during CS presentation. As in previous work on threat-conditioned PSR (Korn et al., 2017), HPR (Castegnetti et al., 2016) and respiratory responses (Castegnetti et al., 2017), we fitted a canonical response function to the difference time series of grand means of scan speed (Fig. 2b). To this end, we used a gamma probability density function with fitted parameters *t*_*0*_ = – 1.90203, *k* = 10.09130, and *θ* = 0.42125 (Fig. 2e). We then used this response function to estimate the amplitude of scan speed responses to CS+ and CS- in a general linear convolution model (Bach et al., 2009). Scan speed response estimates differentiated between CS+ and CS- with an effect size similar to that of scan path length computed over the optimized time window. Results were also very similar when using various different low-pass filters (see Methods).

### Experiment 2: Confirmation

Scan speed for Experiment 2 is shown in Fig. 2c, d. We confirmed that summary statistics derived from Experiment 1 differed between CS+ and CS-. In what follows, we report uncorrected *p* values; all were significant at *p* < .05 after Holm–Bonferroni correction for three tests. Scanpath length, computed over the entire CS-US interval, differed between CS+ and CS- (t(34) = 2.82; *p* = .008; see Table 2 for effect size). Also, scanpath length computed over the optimized time interval differed between CS+ and CS- (t(34) = 4.09; *p* < .001; Fig. 3b). Table 2 shows that the optimal time interval to compute scanpath length was the same as in Experiment 1, such that no further optimization was required. Finally, we computed scan speed responses in a GLM approach, where we shifted the response function by 0.5 s to account for the later US delivery (Fig. 2e) in keeping with the way of computing scanpath length. Estimated responses differed between CS+ and CS- (t(34) = 3.84; *p* < .001). In previous work, we leveraged different CS-US intervals to investigate whether psychophysiological responses were time-locked to CS or US (Castegnetti et al., 2016); given the small (0.5 s) difference in CS presentation time between Experiments 1 and 2 we did not attempt such differentiation here. Overall, it appears that the GLM-based approach did not improve on the effect size to distinguish CS+/CS- in either of Experiments 1–2. This is why we focus on scanpath length computed over the optimized time window as primary measure in the rest of this study.

### Experiment 1–2: Distribution of gaze coordinates and fixation duration

**Fig. 4:**
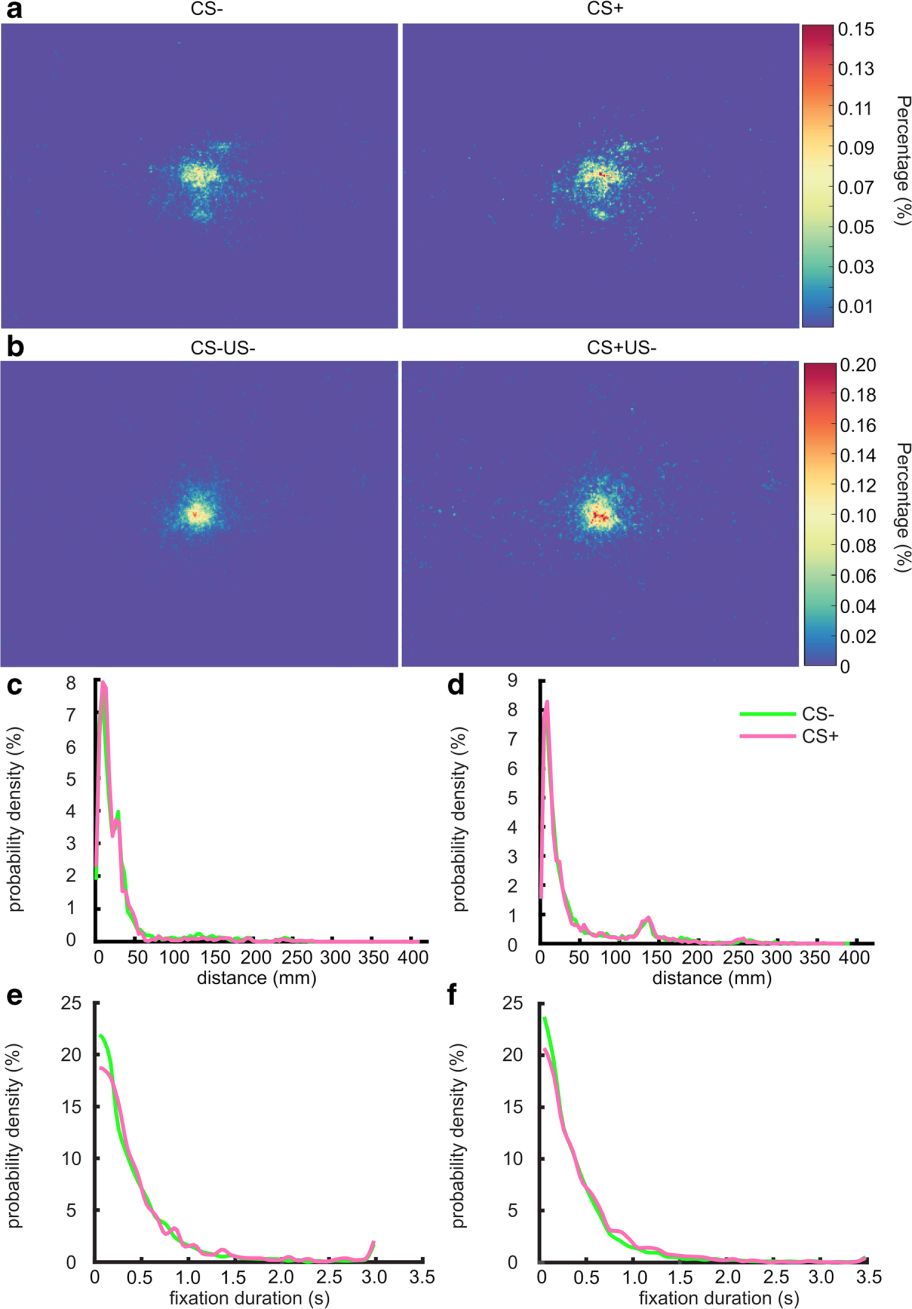
Fixation in Exp 1 and 2. **a**, **b** Fixation distribution on the screen in **a** Exp 1 and **b** Exp 2. Plots are scaled to the real screen with coordinate ranges of [– 156.21, 156.21] mm in *x*-axis and [– 113.445, 113.445] mm in the *y*-axis. **c**, **d** Distribution of distances of fixation points from screen center in **c** Exp 1 and **d** Exp 2. **e**, **f** Distribution of fixation duration in **e** Exp 1 and **f** Exp 2

Next, we sought to explore what causes the difference in scanpath length between CS+ and CS-. We assessed two possible underlying mechanisms: participants scan a wider screen area in CS- trials, and participants saccade more but possibly within the same screen area. Figure 4a, b shows the distribution of gaze coordinates on the screen, where it appears that in both experiments, participants fixated more on the screen center in CS+ than in CS- trials. We computed the distance of fixation points from screen center. An exploratory two-sample Kolmogorov–Smirnov (KS) test showed a significant difference between CS+ and CS- trials in this distance (Exp 1, D = 0.047, p < .001; Exp 2, D = 0.016, p < .001; Fig. 4cd). At the same time, it also appears that participants fixate longer in CS+ than in CS- trials, for both experiments, with significant difference in dwell time distribution between conditions (two-sample KS test: Exp 1, D = 0.054, *p* = .004; Exp 2, D = 0.053, *p* < .001; Fig. 4e, f). Overall, this may imply that the longer scan path in CS- trials results from shorter fixation duration (more scanning) as well as from a wider scanning area.

### Experiment 3: Generalizability to other CS

Experiments 1–2 used monochrome full-screen CS, which may facilitate or inhibit scan speed: spontaneous scanning is unconstrained by visual features, but on the other hand does not reveal any information. Also, Experiments 1–2 were conducted directly after a visually rich instrumental conditioning task where participants might have suspected the occurrence of further visual elements. Here, we sought to generalize our findings to an experiment with visually structured CS and without any other task manipulation. Furthermore, we sought to establish to what extent scan speed responses are extinguished.

During acquisition, scanpath length (computed over the optimal 2-s time period before US onset) discriminated CS+ from CS- (t(25) = 2.23; *p* = .035; *g* = 0.42, paired *t* test, Fig. 3c). Exploratory analysis with the GLM approach confirmed this finding (t(25) = 2.37; *p* = .026; *g* = 0.45, not corrected for multiple comparison).

We then analyzed a subsequent extinction block, during which SCR to CS+ and CS- were not significantly different anymore (t(24) = 1.28; *p* = .21; *g* = 0.25). We observed that HPR were to some extent resistant to extinction (t(25) = 2.49; *p* = .020; *g* = 0.47). Similar to SCR, scanpath length was not different between CS when analyzed over the entire extinction phase (t(25) = 0.01; *p* = .99; *g* < 0.01, Fig. 3d). We then sought to confirm extinction by comparing the second block of acquisition phase with the extinction phase and found a non-significant reduction of the CS+/CS- difference in scan path length from late acquisition to extinction (t(25) = 2.03; *p* = .053; *g* = 0.39).

### Experiment 4: Generalizability to auditory CS during instructed fixation

Next, we sought to investigate to what extent scan speed responses occur in the absence of visual information presented, and when fixation is encouraged by a fixation cross and explicit instruction. In this experiment, we did not observe statistically significant CS+/CS- differences in scanpath length (t(21) = 1.58; *p* = .13; *g* = 0.32, Fig. 3e) or in an exploratory analysis of GLM measures (t(21) = 1.88, *p* = .07, *g* = 0.39).

### Psychometric properties of different threat conditioning measures

**Fig. 5:**
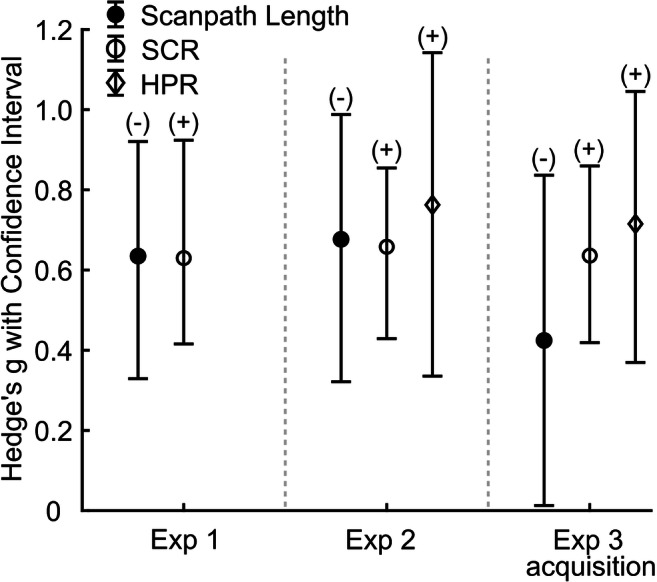
Comparison to other psychophysiological measures. Effect size (Hedge’s *g*) and confidence interval for scanpath length, SCR, and HPR in Experiments 1–3. No HPR data was available for Experiment 1 due to short inter trial interval. *SCR* skin conductance response. *HPR* heart period response. *+/-* direction of the effect (+: measure is larger for CS+, -: measure is larger for CS-). Longer heart period corresponds to bradycardia

Finally, we compare psychometric properties of different threat conditioning measures (Fig. 5). We did not include PSR in this comparison as these are compromised by the lack of an explicit instruction to fixate (Xia et al., 2019a). Our main development criterion was retrodictive validity, which gives an estimate of combined trueness and precision of a measure. Retrodictive validity for scanpath length was similar to SCR, which is one of the most commonly used measures in human threat learning research. Both scan path length and SCR had lower effect size than HPR.

For each measure, our approach provides a single CS-US association estimate per participant, i.e., the difference between CS+ and CS-. If this estimate has the same sign as the average over participants, then the measure correctly classifies CS+ and CS- for this participant. The proportion of participants for which this estimate is positive thus serves as binary classification accuracy. Classification accuracy of scanpath length was 0.81 in Exp 1, 0.74 in Exp 2, and 0.65 in the acquisition phase of Exp 3. This contrasts with 0.76, 0.86, and 0.72 for SCR, and 0.71, 0.80, and 0.77 for HPR, respectively.

To investigate the internal consistency of our measures across trials, we computed the average correlation between consecutive pairs of trials, as surrogate split-half reliability. Reliability was larger for SCR (Exp 1, r = 0.63; Exp 2, r = 0.72; Acquisition phase of Exp 3, r = 0.68) compared to scanpath length (r = 0.41; r = 0.27; r = 0.56) and HPR (Exp 2, r = 0.44; Acquisition of Exp 3, r = 0.64). We further computed the correlation between the different measures, where the CS+/CS- difference for scanpath length is on average negative, and for the other two measures positive. Correlation between the paired CS+/CS- difference in scanpath length and SCR was r = – 0.43 (Exp 1) and r = – 0.11 (Exp 2), between scanpath length and HPR r = 0.13 (Exp 2), and between SCR and HPR r = – 0.05 (Exp 2).

## Discussion

In the current study, we investigated the impact of threat-conditioned cues on summary statistic of gaze patterns, scanpath length, and optimized this measure as an index of human threat conditioning. We first observed shorter scanpath length in CS+ compared to CS- trials when analyzed over the entire CS-US interval. The optimal time window to best differentiate CS+/CS- turned out to be a 2-s time period before anticipated US onset, something that we independently replicated in a second experiment. A GLM-based response measure taking into account the time course of scan speed during CS presentation provided no additional advantage. We confirmed our results in a third experiment with different CS and demonstrated extinction of scanpath length differences when CS+ was no longer reinforced. In a final experiment with auditory CS and instruction to fixate a central screen location, we observed no difference in scanpath length. Thus, it appears that scanpath length differences are not a reflexive response to any CS, but specific either to visual CS or to the absence of an instruction to fixate.

As scanpath length has not been investigated before, we cannot directly compare this study to previous work. However, our results appear consistent with findings on selective overt attention in cue competition paradigms. Since threat-conditioned cues capture bottom-up attention at an early processing stage during visual search (Theeuwes, 2010), one may assume that participants show longer fixation during CS+ trials. This was indeed the case in our study, in line with longer fixation duration on CS+ than on CS- stimuli in cue competition paradigms (Austin & Duka, 2010; Koenig et al., 2017). Interestingly, participants also fixated more on the central part of the full-screen CS and thus scanned an overall smaller area within the visual CS presentation.

As a complementary, but not mutually exclusive interpretation, smaller scanning area and fewer saccades may be conceptually related to freezing as observed during CS+ presentation in rodents.

Freezing behavior is assumed to depend on amygdala-periaqueductal grey (PAG) circuitry in both rodents and humans (Roelofs, 2017). To the best of our knowledge, however, there is currently no evidence showing more freezing (i.e., reduction in body movement) in humans during CS+ than CS- presentation in threat conditioning (see for reviews Hagenaars, Oitzl, & Roelofs, 2014; Roelofs, 2017).

Previous studies demonstrated threat-biased overt attention also after threat conditioning (Mulckhuyse et al., 2013; Schmidt et al., 2015; Hopkins et al., 2016; Mulckhuyse & Dalmaijer, 2016; Koenig et al., 2017; Nissens et al., 2017). Here, we found that the CS+/CS- difference was reduced from acquisition to extinction, and there was no significant difference of scanpath length between CS+/CS- during threat extinction any more, as for SCR. This discrepancy with previous literature could possibly be due to weaker conditioning in the current study. Experiments with higher reinforcement rates during acquisition may shed light on this point.

During an auditory threat conditioning experiment with instruction to fixate a central fixation cross, we found no difference in scanpath length between CS+ and CS-. This is in line with a salience-based account of scanpath length, whereby saliency of binaurally presented auditory information should not spatially bias visual attention. However, we note that the presence of a fixation cross during CS, and the instruction to fixate on this cross, may also have suppressed possible gaze differences between CS+/CS-.

Split-half reliability of the scanpath measure was moderate, and smaller than for SCR, although retrodictive validity was similar. As a possible reason, retrodictive validity simultaneously depends on trueness and precision of a measure, whereas reliability only depends on precision. For two measures with similar retrodictive validity, the more reliable one will have higher precision and lower trueness, compared the less reliable one (Bach et al., 2020). It is thus possible that scanpath length provides higher trueness but lower precision to measure CS-US association, compared to SCR. As a second possible reason, different threat conditioning measures may not index the same learning quantity (Tzovara et al., 2018; Bach, Tzovara, & Vunder, 2018b; Ojala & Bach, 2020), and although they may similarly distinguish CS+/CS- when averaged over trials, their trial-by-trial dynamics can be different. Our method of approximating internal consistency is susceptible to trial-by-trial changes in the learning measure. If, for example, one measure asymptotes more quickly than another, its reliability will appear higher, even if the measure is not more precise when averaged over trials.

In keeping with such differences between measures, the correlation between averaged scanpath length and SCR was moderate, and almost zero between scanpath length and HPR. This may mean that these metrics may not measure the same latent variable even on average, although our small sample size precludes any strong conclusions at this point. Furthermore, this correlation as well as reliability depend on interindividual differences in learning. Experimental tasks such as the one used here seek to minimize individual differences (Hedge et al., 2018), and may thus not be optimally suited to assess reliability and the correlation between measures.

Our work has several limitations. First, in order to maintain attention during experiments, participants were asked to press designated keys to respond to CS presentations. Although we used full-screen colors and patterns as visual cues, this instruction might restrict voluntary visual search during CS presentation and could be an additional interference on threat driven attentional bias, waiting for future exploration. We did not examine how threat memory retention during early extinction trials is reflected in scanpath length, which could be further studied in future work with larger sample sizes. Finally, we compared scanpath length against SCR and HPR only. These measures have moderate retrodictive validity (Bach & Melinscak, 2020), but this may depend on the CS-US interval, which was rather short here (3–3.5 s), precluding strong conclusions from this comparison.

We developed this threat conditioning measure with an eye on concurrent pupil size recordings but we note that the lack of a fixation cross may decrease retrodictive validity of pupillary responses (Xia et al., 2019a). In order to combine both measures, more sophisticated correction of the pupil foreshortening error may be required (Hayes & Petrov, 2016). On the other hand, scanpath length could be conceived as an alternative to pupillary responses in situations of low-quality eye tracking, because recording gaze coordinates likely requires lower camera resolution than pupillary responses and has been reported using consumer-grade cameras (Papoutsaki, Laskey, & Huang, 2017; Papoutsaki et al., 2016). With the resolution of such cameras, typical threat-conditioned PSR are on the order of one pixel and thus possibly not detectable at all.

In conclusion, the present study investigated a summary statistic of gaze patterns, scanpath length, as a potential measure of threat conditioning. The effect size for scanpath length in our study was comparable to that of SCR, a commonly used threat conditioning measure (Staib et al., 2015). In addition to being a potential complement to existing measures, gaze patterns have a potential to be recorded with consumer-grade hardware or in other species (Hannula et al., 2010). Thus, we hope to have contributed to the toolkit of comparative and translational threat conditioning research in humans.

### Author note

Yanfang Xia, Computational Psychiatry Research, Department of Psychiatry, Psychotherapy, and Psychosomatics; Psychiatric Hospital; University of Zurich, 8032 Zurich, Switzerland. Neuroscience Center Zurich; University of Zurich, 8057 Zurich, Switzerland. Filip Melinscak, Computational Psychiatry Research, Department of Psychiatry, Psychotherapy, and Psychosomatics; Psychiatric Hospital; University of Zurich, 8032 Zurich, Switzerland. Neuroscience Center Zurich; University of Zurich, 8057 Zurich, Switzerland. Dominik R. Bach, Computational Psychiatry Research, Department of Psychiatry, Psychotherapy, and Psychosomatics; Psychiatric Hospital; University of Zurich, 8032 Zurich, Switzerland. Neuroscience Center Zurich; University of Zurich, 8057 Zurich, Switzerland. Wellcome Centre for Human Neuroimaging and Max Planck/UCL Centre for Computational Psychiatry and Ageing Research, University College London, London WC1 3BG, UK.

The authors thank Dongqi Bao for help in data collection and Samuel Gerster for technical support. This work was supported by the University of Zurich’s Clinical Research Priority Program for the CRPP “Synapse & Trauma”. DRB is supported by funding from the European Research Council (ERC) under the European Union’s Horizon 2020 research and innovation programme (Grant agreement No. ERC-2018 CoG-816564 ActionContraThreat). The Wellcome Centre for Human Neuroimaging is funded by a strategic grant from the Wellcome Trust (205103/Z/16/Z).

### Open practices statement

All data sets are available in anonymized form on www.zenodo.org (Table 1). Analysis code is available from OSF (10.17605/OSF.IO/U4GRC). All data processing methods are implemented in the most recent version of PsPM (https://github.com/bachlab/PsPM/releases). None of the experiments was preregistered.
